# Desenho de Estudo de um Estudo Observacional Brasileiro sobre o uso de Edoxabana em Pacientes com Fibrilação Atrial (EdoBRA)

**DOI:** 10.36660/abc.20230392

**Published:** 2024-04-12

**Authors:** Dalton Bertolim Précoma, Rafael Paletta da Silva, Allyson Nakamoto, Viviane Mariz Omar, Danilo Lopes, José Francisco Kerr Saraiva

**Affiliations:** 1 Hospital Angelina Caron Curitiba PR Brasil Hospital Angelina Caron, Curitiba, PR – Brasil; 2 Daiichi Sankyo Brasil Farmacêutica São Paulo SP Brasil Daiichi Sankyo Brasil Farmacêutica, São Paulo, SP – Brasil; 3 Pontifícia Universidade Católica de Campinas Campinas SP Brasil Pontifícia Universidade Católica de Campinas, Campinas, SP – Brasil

**Keywords:** Anticoagulantes Orais, Edoxabana, Estudo Observacional, Segurança, Eficácia

## Abstract

**Fundamento:**

Os ensaios clínicos demonstraram a segurança da Edoxabana, um anticoagulante oral não dependente de vitamina K (NOAC), e a sua eficácia na prevenção de acidente vascular cerebral e embolia sistémica em pacientes com fibrilação atrial não valvar (FANV) e também na prevenção e tratamento de tromboembolismo venoso. No entanto, pesquisas adicionais são necessárias para avaliar a segurança e a eficácia da Edoxabana em um cenário real na população brasileira.

**Objetivo:**

A fim de compreender os riscos e benefícios do uso da Edoxabana em cenários clínicos de rotina, o estudo EdoBRA está sendo conduzido para obter informações sobre a segurança e eficácia do uso da Edoxabana em pacientes não pré-selecionados com FANV no Brasil.

**Métodos:**

O estudo EdoBRA é um estudo multicêntrico, prospectivo e observacional, realizado em 36 centros no Brasil. São elegíveis para este estudo pacientes com FANV, ≥ 18 anos de idade, tratados com Edoxabana disponível comercialmente, que iniciaram o tratamento por pelo menos 14 dias e não mais do que 90 dias antes da data de inclusão no estudo, e que não estão participando de nenhum outro estudo de intervenção. Ao todo, 700 pacientes devem ser inscritos e acompanhados por um ano, com coletas de dados programadas para o período basal e 3, 6 e 12 meses após a inscrição no estudo. O objetivo primário de segurança é o sangramento clinicamente relevante (de acordo com critérios da Sociedade Internacional de Trombose e Hemostasia - ISTH), e o objetivo secundário de eficácia são desfechos cardiovasculares relevantes relacionados à FANV.

**Conclusão:**

O estudo observacional EdoBRA gerará informações adicionais relevantes sobre a Edoxabana enquanto NOAC em diversos aspectos do manejo de pacientes no atendimento clínico de rotina, como perfil de segurança e efetividade em pacientes com FANV no Brasil.

**Figure:**
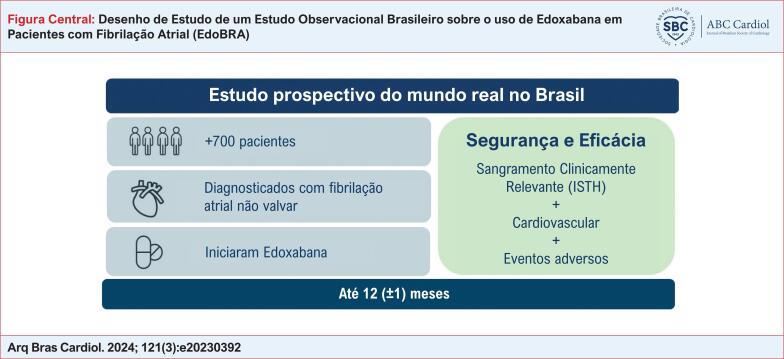


## Introdução

A fibrilação atrial (FA) é a arritmia mais comum relatada na prática clínica, afetando aproximadamente 2,5% dos adultos com 20 anos ou mais.^1 , 2^ Como em outras partes do mundo, na América Latina há uma prevalência crescente de FA em idosos, caracterizada por uma forma silenciosa, até a primeira detecção, até uma forma persistente de longa duração até a forma permanente.^2 – 4^ A maioria dos casos é FA não valvar (FANV), que geralmente está associada ao aumento da morbidade e mortalidade. Os principais problemas associados são: risco de acidente vascular cerebral, sangramento e morte por causas cardiovasculares.^5 , 6^ As hospitalizações devido à FA ou suas complicações (agravamento da insuficiência cardíaca, complicações tromboembólicas e manejo de arritmias agudas) representam de um terço a dois terços de todas as internações por arritmias cardíacas.^7 – 9^ Pacientes com FANV apresentam qualidade de vida significativamente pior em comparação com controles saudáveis, a população em geral ou pacientes com doença coronariana.^10^ O risco anual de acidente vascular cerebral é de cinco a seis vezes maior em pacientes com FA do que em pessoas com ritmo cardíaco normal.^11^

O objetivo do tratamento da FANV com anticoagulante é prevenir acidente vascular cerebral e/ou embolia sistêmica.^12 , 13^ Contudo, uma complicação importante da terapia anticoagulante é o sangramento, que também pode influenciar a adesão e persistência do paciente. Desta forma, diferentes anticoagulantes, como os anticoagulantes orais não dependentes de vitamina K (NOAC), foram desenvolvidos para tratar pacientes com FANV e solucionar essa questão. Os NOACs permitem a administração de doses fixas sem a necessidade de monitoramento rotineiro da coagulação, de ajustes de dose e menor risco de sangramento, conforme comumente descrito no uso de anticoagulantes mais antigos, como a varfarina. Atualmente, quatro NOACs foram aprovados por diversas autoridades regulatórias em todo o mundo, incluindo a Agência Nacional de Vigilância Sanitária (ANVISA), e estão disponíveis para uso clínico: inibidor direto da trombina, dabigatrana; e os inibidores orais do fator Xa, rivaroxabana, apixabana e edoxabana. Esses novos anticoagulantes orais são tão seguros e eficazes quanto a varfarina na prevenção de acidente vascular cerebral e embolia sistêmica em pacientes com FA^14 – 17^ e seu uso clínico rotineiro foi descrito na literatura.^18^

A maioria dos ensaios clínicos randomizados adotou critérios de elegibilidade restritos, excluindo explicitamente pacientes ou inscrevendo apenas participantes relativamente saudáveis, com menos comorbidades ou comprometimentos funcionais. Assim, registros prospectivos de evidências do mundo real podem fornecer informações relevantes sobre a eficácia desses medicamentos na população brasileira. O estudo EdoBRA foi desenhado para coletar prospectivamente informações detalhadas sobre o uso de Edoxabana no cenário clínico real, em pacientes com FANV não pré-selecionados no Brasil. Dados do mundo real nos permitiriam uma maior compreensão das possibilidades de tratamento da FANV e ampliariam nossa perspectiva sobre a população de pacientes.

### Edoxabana

A Edoxabana é um anticoagulante administrado por via oral que inibe o fator de coagulação Xa. Foi aprovado para comercialização no Brasil pela ANVISA em março de 2018 para as seguintes indicações: redução do risco de acidente vascular cerebral e de eventos embólicos sistêmicos (EES) em pacientes adultos com FANV e para tratamento de tromboembolismo venoso (TEV), incluindo trombose venosa profunda (TVP) e embolia pulmonar (EP), e a prevenção de TEV recorrente (TVP e/ou EP).

Sua aprovação teve como base os resultados do estudo ENGAGE AF-TIMI 48, um estudo multinacional, randomizado, duplo-cego, duplo simulado, de não inferioridade, que comparou a eficácia e a segurança de dois regimes posológicos de Edoxabana uma vez ao dia com varfarina com dose ajustada (razão normalizada internacional desejada [RNI] 2,0 a 3,0) em pacientes adultos (N = 21.105) com FANV com risco moderado a alto de acidente vascular cerebral. A Edoxabana, tanto em dose alta (60/30 mg) quanto em dose baixa (30/15 mg), uma vez ao dia, apresentou eficácia semelhante na prevenção de acidente vascular cerebral ou EES e taxas significativamente mais baixas de sangramento e morte por causas cardiovasculares em comparação com a varfarina bem administrada em pacientes com FA.^17 , 19^

O estudo Hokusai-VTE (N = 8.292) também foi um estudo fundamental para a aprovação da Edoxabana. O estudo constatou que os pacientes com TEV sintomático tiveram uma duração de tratamento flexível de 3 a 12 meses e descobriu que, após a heparina inicial, a dose de 60 mg de Edoxabana uma vez ao dia não foi inferior à varfarina com dose ajustada (RNI 2,0 a 3,0) para a prevenção de TEV recorrente, e também apresentou um risco significativamente menor para o composto de sangramentos graves ou não graves mas clinicamente relevantes (desfecho primário de segurança).^20 , 21^

Os resultados de outro estudo fundamental são do estudo ELDERCARE-AF (N = 984). O objetivo foi avaliar a prevenção de acidente vascular cerebral em pacientes bastante idosos com FA. Após este estudo multicêntrico, randomizado, duplo-cego, controlado por placebo e orientado a eventos, os pesquisadores descobriram que uma dose única diária de 15 mg de Edoxabana foi superior ao placebo na prevenção de acidente vascular cerebral ou embolia sistêmica (2,3% em Edoxabana e 6,7 % no placebo) e não resultou em uma incidência significativamente maior de sangramentos graves quando comparado ao placebo.^22^

Um grande estudo europeu com dados reais, que relatou os desfechos ao longo de dois anos do tratamento com Edoxabana, não encontrou sinais de segurança e/ou taxas de eventos adicionais além daqueles observados no ETNA-AF após um ano e no estudo ENGAGE AF-TIMI 48. Estes desfechos reforçam os dados encontrados nos ensaios clínicos realizados sobre Edoxabana e as recomendações de aprovação em um cenário real. Além disso, estudos comparativos do mundo real indicaram melhores achados de eficácia em pacientes tratados com Edoxabana do que outras terapias, como femprocumona ou até mesmo outros NOACs.^23^

Desde sua disponibilização no Brasil, milhares de pacientes foram tratados com esse NOAC, mas também é importante avaliar seu desempenho em um cenário clínico de rotina na população brasileira. No presente artigo, descrevemos o desenho e a metodologia do estudo EdoBRA, incluindo os parâmetros comuns (ou seja, os chamados "dados principais"), os períodos de observação e outros detalhes técnicos, como a consistência da terminologia.

Para ajudar a compreender os riscos e benefícios do uso de Edoxabana em um cenário clínico próximo à prática clínica regular, realizamos este estudo não intervencional (NI) para obter informações sobre os aspectos de segurança e eficácia em pacientes não pré-selecionados com FANV, tratados com Edoxabana no Brasil.

O objetivo principal deste estudo é avaliar a segurança de Edoxabana ao longo de um ano em relação à ocorrência de sangramento clinicamente relevante (sangramento clinicamente relevante não grave e sangramento grave) em pacientes com FANV. Espera-se também que os dados coletados durante o estudo ofereçam uma visão geral dos desfechos de eficácia na prevenção de acidentes vasculares cerebrais (isquêmicos e hemorrágicos), EES, ataque isquêmico transitório (AIT), eventos cardiovasculares adversos graves (MACE, *endpoint* composto de IM não fatal, acidente vascular cerebral não fatal, EES não fatal e morte por causa cardiovascular ou sangramento), TEV, síndrome coronariana aguda (SCA) e hospitalizações relacionadas a condições cardiovasculares (CV).

Desta forma, evidências do mundo real na prática clínica de rotina sobre o uso da Edoxabana por até um ano serão coletadas e avaliadas em 700 pacientes com FANV, tratados por médicos especializados e não especializados em diferentes centros hospitalares no Brasil.

## Métodos

### Desenho do Estudo

O estudo EdoBRA, patrocinado pela Daiichi-Sankyo Brasil, é um estudo multicêntrico, prospectivo e observacional (NI) realizado no Brasil. Foi desenhado como um estudo NI por incluir pacientes que receberam prescrição de Edoxabana sem qualquer influência na prescrição para inclusão no estudo. Os investigadores principais não podem incluir pacientes aos quais prescreveram Edoxabana; apenas são elegíveis pacientes encaminhados por outros médicos.

**Figura 1:**
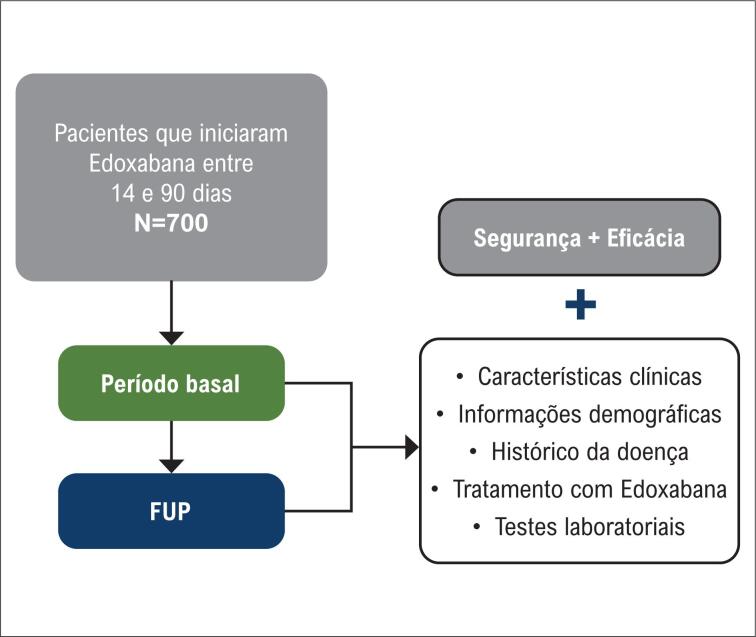
Fluxograma de acompanhamento dos pacientes. FUP: acompanhamento. Declaração de Isenção de Responsabilidade: Por se tratar de um estudo não intervencional no mundo real, as visitas não são agendadas. Apenas os dados de uma prática clínica de rotina serão documentados.

A inscrição no estudo e a coleta de dados começaram em outubro de 2019 e foram concluídas em fevereiro de 2023. Espera-se documentar os dados da prática clínica de rotina em quatro momentos diferentes: No período basal, e após 3 (±2), 6 (±2) e 12 (±1) meses da inclusão no estudo (um ano de acompanhamento). Até mesmo os pacientes que descontinuarem o tratamento com Edoxabana serão acompanhados por até um ano. As visitas dos pacientes são realizadas de acordo com o atendimento clínico regular e não são influenciadas pelo momento previsto para documentação dos dados. Por ser um estudo observacional, não foi necessário um cronograma de visitas específico. Desta forma, a frequência da coleta de dados, na maioria das vezes, coincide com as consultas clínicas regulares de pacientes com FANV aos serviços de saúde, de acordo com a prática clínica. O desenho do estudo é apresentado na Figura 1 .

### Critérios de elegibilidade e exclusão

O estudo EdoBRA pretendeu incluir aproximadamente 700 pacientes com diagnóstico estabelecido de FANV (todos os tipos de FANV foram considerados), tratados com Edoxabana de acordo com a bula local. Os pacientes elegíveis possuíam ≥ 18 anos, apresentavam diagnóstico de FANV e iniciaram o tratamento com Edoxabana por pelo menos 14 dias e não mais do que 90 dias antes da inclusão no estudo. Os pacientes devem consentir em participar e não devem estar participando de nenhum outro estudo intervencional. No entanto, é permitida participação simultânea em qualquer outro estudo/registro NI. Nenhum outro critério de exclusão explícito foi adotado para minimizar o viés de seleção e permitir a máxima documentação da prática clínica de rotina neste estudo NI.

### Avaliações

O objetivo principal deste estudo é avaliar a segurança da Edoxabana em um cenário de atendimento clínico regular. O perfil de segurança será avaliado pela frequência de sangramento grave ou sangramento não grave clinicamente relevante (CRNM) de acordo com os critérios da Sociedade Internacional de Trombose e Hemostasia (ISTH) em pacientes com FANV durante um ano de acompanhamento.

Um evento hemorrágico clinicamente evidente (ou seja, sangramento visualizado por exame ou imagem radiológica) que atende a pelo menos um dos seguintes critérios: a) sangramento fatal; b) sangramento sintomático em área ou órgão crítico, como retroperitoneal, intracraniana, intraocular, intraespinhal, intra-articular, pericárdica ou intramuscular com síndrome compartimental.

Com base na classificação da ISTH, um evento hemorrágico não grave, mas clinicamente relevante (CRNM) foi definido de acordo com os seguintes pressupostos: sangramento excessivo que requer intervenção médica de um profissional de saúde; sangramento que exige hospitalização ou aumento do nível de cuidados; sangramento que exige uma avaliação presencial (ou seja, não apenas por telefone ou comunicação eletrônica), mas que não atenda aos critérios para um evento hemorrágico grave.

**Figura 2:**
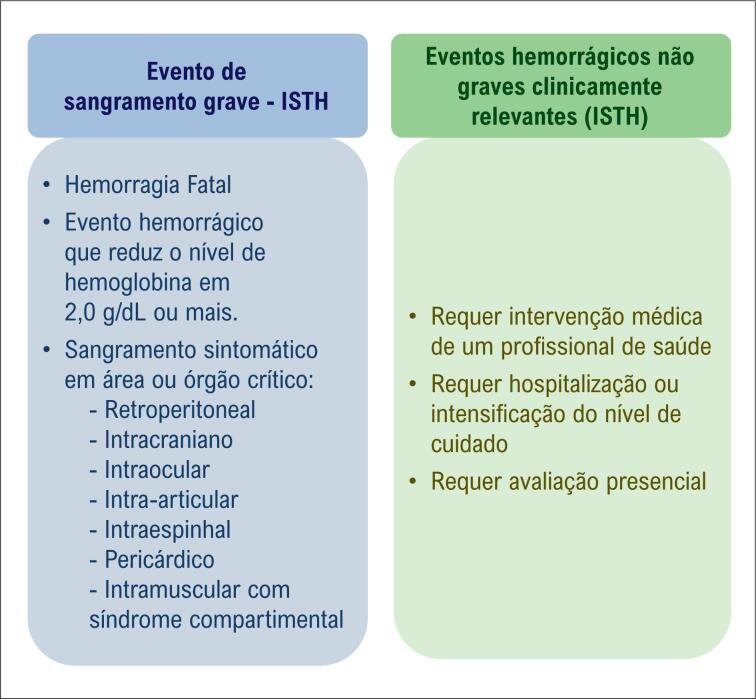
Eventos que definem eventos hemorrágicos graves (conforme ISTH) e eventos hemorrágicos não graves clinicamente relevantes (conforme ISTH). ISTH: Sociedade Internacional de Trombose e Hemostasia.

Caso os pacientes apresentem outros eventos hemorrágicos evidentes que não atendam aos critérios de um evento hemorrágico grave ou um evento hemorrágico não grave clinicamente relevante (por exemplo, epistaxe que não requer atendimento médico), tais eventos devem ser classificados como eventos hemorrágicos menores. Além disso, todos os eventos hemorrágicos detectados durante a coleta de dados devem ser avaliados de acordo com as definições de sangramento da ISTH.^24 , 25^ Se o paciente apresentar outros eventos, como redução dos níveis de hemoglobina sem sangramento, deve ser considerado um evento não hemorrágico. A diferença entre a classificação dos eventos hemorrágicos ISTH está descrita na Figura 2 .

O objetivo secundário inclui a eficácia da Edoxabana em evitar eventos como acidentes vasculares cerebrais (isquêmicos e hemorrágicos), EES, AIT, MACE, TEV, SCA, *endpoint* composto de eventos não fatais - MACE (IM não fatal, acidente vascular cerebral não fatal, EES não fatal e morte por causa cardiovascular ou sangramento) e causas de hospitalização (de modo geral, relacionadas à condição cardiovascular ou relacionadas à condição hemorrágica). Por fim, também será avaliado o perfil de segurança em relação a outros eventos adversos que não atendem aos critérios de desfecho primário.

Assim, todas as avaliações de segurança que ocorrerem entre a inclusão no estudo e o acompanhamento final serão registradas, incluindo todos os eventos adversos. Além disso, as características demográficas e clínicas no período basal serão registradas para todos os pacientes. O histórico e diagnóstico FA, detalhes das informações de prescrição de Edoxabana, procedimentos diagnósticos/terapêuticos e desfechos clínicos serão documentados no período basal e atualizados com quaisquer novas informações para cada procedimento registrado no período de até um ano (±1 mês).

### Gestão de dados

Todos os dados serão coletados a partir de informações registradas rotineiramente nas fichas/nos prontuários dos pacientes. Não há recomendações para alterar a prática clínica rotineira, devido a este estudo NI. Nenhuma visita ou exame específico, testes laboratoriais ou procedimentos são obrigatórios como parte deste estudo. Uma vez que nem o investigador principal nem os demais investigadores poderão incluir pacientes aos quais prescreveram Edoxabana, a equipe do estudo será a principal responsável pela coleta de dados dos pacientes encaminhados. O acesso aos documentos de origem (por exemplo, registros médicos) é obrigatório para a coleta de dados no prontuário eletrônico (eCRF). Os dados de origem também devem estar disponíveis para atividades de monitoramento do centro.

Um sistema *Eletronic Data Capture* (EDC - Ficha clínica eletrônica) é utilizado para a coleta dos dados. Todos os dados clínicos são documentados por meio de uma ferramenta de coleta eletrônica de dados clínicos (eCRF), criada especificamente para este estudo. São utilizadas variáveis e definições predefinidas e o preenchimento de campos de texto livre foi limitado tanto quanto possível. Na fase inicial do estudo, foi criado um plano de gestão de dados que descreve todo o processo de coleta de dados. São realizadas verificações automáticas de plausibilidade e revisão manual dos dados no momento da inserção dos dados para evitar possíveis erros e para permitir correções ou confirmação pelos centros. Além disso, o eCRF também possui um sistema automático de notificação de eventos adversos. Para atender aos prazos de notificação de eventos adversos, o eCRF envia uma mensagem automática para os monitores e a equipe de farmacovigilância sempre que o coletor de dados insere uma informação relacionada a um evento adverso grave ou a uma reação adversa a um medicamento.

Histórico médico, eventos clínicos e descrições e eventos adversos/reações medicamentosas serão codificados usando o Dicionário Médico para Atividades Regulatórias (MedDRA). Os eventos clínicos são baseados no diagnóstico e na avaliação do médico. Os medicamentos serão codificados usando o Dicionário de Medicamentos da Organização Mundial da Saúde Aprimorado (WHO-DDE).

O número de casos faltantes será informado para cada variável de interesse na análise. O fluxo de participantes do estudo durante todo o período do estudo será caracterizado em termos do número de participantes que completaram o estudo, desistiram dele (com a descrição dos motivos) ou faleceram. Adicionalmente, se o tratamento de um paciente com Edoxabana for permanentemente descontinuado por qualquer motivo, a qualquer momento após o ponto de coleta de dados do período basal, o investigador precisará documentar o fato no eCRF adequadamente.

Todos os eventos adversos graves (EAGs) e todas as reações adversas a medicamentos (RAMs) devem ser inseridos na respectiva seção de coleta de dados do eCRF assim que o médico tiver conhecimento. Eventos adversos não graves que não estejam relacionados à Edoxabana não serão documentados. Os eventos adversos serão relatados de acordo com a exigência nacional e a legislação brasileira.

### Tamanho da amostra e análise estatística

Nenhuma consideração formal do tamanho da amostra foi realizada para este estudo. O estudo pretende inscrever 700 pacientes em 36 centros. É razoável estimar que 2-9% dos pacientes apresentarão pelo menos um evento hemorrágico clinicamente relevante durante o acompanhamento, o que permitirá a realização de análises descritivas e estimativas de incidência de sangramento.

Como este estudo é descritivo e observacional, não se pretende testar hipóteses estatísticas. Portanto, apenas estatísticas descritivas serão realizadas para descrever todos os dados coletados, e geralmente limitadas a tabelas de frequência ou estatísticas resumidas (por exemplo, médias ± desvio padrão ou medianas ± quartis), por exemplo, para dados demográficos. A incidência de eventos hemorrágicos graves e sangramento não grave clinicamente relevante será calculada com base na proporção de pacientes que apresentam eventos hemorrágicos no período de acompanhamento. As análises dos dados de acompanhamento são planejadas aos três e seis meses, seguidas pela análise do período basal e as análises finais dos dados de acompanhamento completos até um ano (±1 mês). O número de participantes com pelo menos um evento será apresentado (n [%]) separadamente para cada tipo de evento clínico. As reações adversas aos medicamentos serão resumidas com base no MedDRA.

Além disso, quando aplicável, a data índice para as análises do estudo será a data de início de Edoxabana. Todas as análises estatísticas serão realizadas utilizando Python versão 3.6.9.

### Controle de qualidade

Este estudo foi realizado de acordo com as regras das "Boas Práticas Farmacoepidemiológicas" (BPFE) e regulamentos locais relevantes. Mecanismos de controle de qualidade relacionados foram devidamente empregados.

O monitoramento no centro será realizado em 30% dos pacientes selecionados aleatoriamente, no mínimo. Durante o monitoramento no centro, o monitor verificará 100% da documentação de consentimento informado e realizará a verificação dos dados de origem em relação aos registros médicos dos pacientes selecionados. Durante as atividades de monitoramento, será dada especial atenção à integralidade e exatidão dos dados de segurança. A validação e o controle de qualidade serão realizados para garantir que os dados relatados sejam precisos e completos, na medida do possível, em um cenário de atendimento clínico de rotina e que a realização do estudo EdoBRA siga o planejamento e atenda às exigências regulatórias locais aplicáveis.

## Discussão

A investigação de observação tem um papel fundamental na confirmação da eficácia e segurança das intervenções recentemente aprovadas em uma ampla variedade de pacientes com características diferentes (dados demográficos, comorbilidades, acesso à assistência médica, entre outros), tratados em um cenário clínico em que o controle da terapia do paciente frequentemente não é tão rigoroso quanto em um ensaio clínico. Também podem ser identificados efeitos adversos associados ao uso de produtos farmacêuticos que não foram previstos com base em pesquisas realizadas para fundamentar o processo de aprovação de medicamentos. O estudo EdoBRA é um estudo multicêntrico, prospectivo, observacional (NI) realizado no Brasil para avaliar a segurança e eficácia da Edoxabana em pacientes com FANV. O desenho dos dados principais do estudo EdoBRA aborda questões importantes sobre o uso clínico da Edoxabana, oferecendo evidências das características clínicas e desfechos importantes no uso clínico de rotina, permitindo complementar as informações obtidas no estudo clínico randomizado já publicado.^26^

Anteriormente, alguns ensaios clínicos randomizados foram realizados para avaliar o risco de sangramento. O ensaio ENGAGE AF-TIMI 48,^17^ por exemplo, foi um grande estudo realizado com 21.105 participantes com risco de FA moderado a alto que comparou Edoxabana com Varfarina. Nos participantes que receberam Edoxabana, foram observadas taxas significativamente mais baixas de sangramento e morte por causas cardiovasculares. Em consonância com o estudo ENGAGE, outros estudos observaram taxas menores de sangramento em pacientes com TEV agudo^27^ ou FANV.^28^ Além disso, outros estudos clínicos randomizados nos quais os participantes receberam 30 e 60 mg de Edoxabana, bem como uma meta-análise agrupada de cinco estudos clínicos randomizados e prospectivos totalizando 311.262 pacientes, indicaram que a Edoxabana parece ter um perfil de segurança favorável em comparação à varfarina em relação ao risco de sangramento e mortalidade.^29 – 31^ Embora a Edoxabana tenha apresentado um bom desempenho nesses estudos, existem preocupações sobre o potencial de sangramento na prática clínica que precisam ser investigadas com estudos do mundo real.^31^ Embora estudos clínicos randomizados sejam o modelo padrão-ouro para demonstrar a segurança e eficácia de um novo medicamento, os critérios de inclusão e exclusão nesses casos selecionam determinados pacientes e não representam toda a população. Considerando as limitações naturais dos estudos clínicos randomizados, as agências reguladoras de todo o mundo entendem que os estudos não intervencionais complementam as informações obtidas nos estudos randomizados.^32^

Alguns estudos observacionais sobre a Edoxabana foram desenvolvidos nos últimos anos. O Estudo STATES^33^ avaliou a ocorrência de sangramentos sintomáticos decorrentes de acidente vascular cerebral cardioembólico agudo em pacientes que receberam tratamento precoce com Edoxabana em uma pequena população idosa (n = 75) durante três meses. Os desfechos apontaram a Edoxabana como segura, mas recomendam mais estudos com uma população maior e um período de acompanhamento mais longo. Um grande estudo observacional e prospectivo – ETNA-AF, realizado em dez países europeus avaliou a segurança e eficácia de 30 mg e 60 mg de Edoxabana nos cuidados de rotina de 13.092 pacientes durante um ano. Concluíram que a taxa de acidente vascular cerebral, embolia sistêmica (0,82%/ano) e sangramento grave (1,05%/ano) foi baixa na coorte não selecionada,^34^ reforçando as evidências de segurança de Edoxabana. Ademais, foi publicado recentemente um período de acompanhamento de dois anos do ETNA-AF, oferecendo evidências reais da segurança e eficácia em longo prazo da Edoxabana. Os desfechos reforçam as baixas taxas de acidente vascular cerebral (0,70%) e sangramentos graves (0,97%) em pacientes com FA e apontam uma alta adesão ao tratamento com quase 70% dos pacientes em tratamento com Edoxabana em dois anos de acompanhamento.^35^ A análise dos preditores de risco ajustados por idade mostrou que a história no período basal, como AIT e depuração de creatinina estimada recalculada, foi o preditor mais forte de acidente vascular cerebral isquêmico e sangramento grave, respectivamente. Além dessas sólidas evidências, os dados sobre sua efetividade e segurança no atendimento clínico de rotina ainda são limitados na população brasileira.^35^ Portanto, estudos realizados após a autorização de comercialização, como o EdoBRA, podem contribuir com informações adicionais importantes sobre aspectos reais do manejo de pacientes no atendimento de rotina no Brasil.

O presente estudo específico desempenha um papel importante e único na avaliação da eficácia e segurança no mundo real, no atendimento clínico regular, avaliando a ocorrência de sangramento clinicamente relevante (conforme ISTH) durante um ano de acompanhamento em aproximadamente 700 pacientes no Brasil. Podem ser examinadas questões relativas à existência de um padrão de uso regional e/ou características dos pacientes, como isso afeta os desfechos e o momento dos eventos clínicos relativos ao início da dosagem, interrupção da dosagem ou qualquer situação relacionada ao esquema de tratamento. Além disso, deve-se considerar que a população brasileira é caracterizada por uma grande diversidade étnica, com pessoas que descendem de duas ou mais etnias diferentes, e isso não é facilmente encontrado em outras regiões do mundo,^36^ o que também justifica um melhor conhecimento do comportamento dos anticoagulantes por meio de registros da vida real.

Como este estudo visa coletar e avaliar evidências do mundo real, é necessário considerar algumas limitações comuns aos estudos não intervencionais e outros aspectos. A investigação observacional eficaz deve reconhecer o potencial de enviesamento e tentar minimizá-lo tanto no desenho quanto na análise, bem como descrever com precisão as limitações. A falta de randomização pode gerar um viés de seleção especialmente na comparação de desfechos. Como o estudo não é intervencional, apenas dados do tratamento clínico de rotina podem ser obtidos. Portanto, algumas informações podem estar indisponíveis ou faltando. O desenho de estudo escolhido para este estudo não permite conclusões de causalidade, mas sim interferências e indicação de caminhos para futuras investigações. Como resultado da natureza descritiva do estudo, os resultados devem ser interpretados com cuidado para evitar comparações diretas entre diferentes grupos. Embora os centros selecionados sejam localizados em diferentes regiões do Brasil, a maioria deles se concentra nas regiões sudeste, nordeste e sul do país. Isso pode reduzir a representatividade da população brasileira.

## Conclusão

O EdoBRA fornecerá um conjunto abrangente de dados para fundamentar a segurança e a utilidade clínica da Edoxabana em pacientes com FA na prática de rotina. Adicionalmente, o estudo proporcionará informações importantes sobre a gestão de eventos adversos que ocorrem em pacientes tratados com Edoxabana, bem como indicadores que podem contribuir para maximizar a satisfação do paciente e melhorar os desfechos no mundo real.

## References

[1] Le Heuzey JY, Otmani A, Marijon E, Waintraub X, Lepillier A, Chachoua K (2008). Atrial Fibrillation: The Most Common Arrhythmia. Presse Med.

[2] Hindricks G, Potpara T, Dagres N, Arbelo E, Bax JJ, Blomström-Lundqvist C (2021). 2020 ESC Guidelines for the Diagnosis and Management of Atrial Fibrillation Developed in Collaboration with the European Association for Cardio-Thoracic Surgery (EACTS): The Task Force for the Diagnosis and Management of Atrial Fibrillation of the European Society of Cardiology (ESC) Developed with the Special Contribution of the European Heart Rhythm Association (EHRA) of the ESC. Eur Heart J.

[3] Mostaza JM, Suarez C, Cepeda JM, Manzano L, Sánchez D, PERFILAR study investigators (2021). Demographic, Clinical, and Functional Determinants of Antithrombotic Treatment in Patients with Nonvalvular Atrial Fibrillation. BMC Cardiovasc Disord.

[4] Cubillos L, Haddad A, Kuznik A, Mould-Quevedo J (2014). Burden of Disease from Atrial Fibrillation in Adults from Seven Countries in Latin America. Int J Gen Med.

[5] Magalhães LP, Figueiredo MJO, Cintra FD, Saad EB, Kuniyishi RR, Teixeira RA (2016). II Diretrizes Brasileiras de Fibrilação Atrial. Arq Bras Cardiol.

[6] Massaro AR, Lip GYH (2016). Stroke Prevention in Atrial Fibrillation: Focus on Latin America. Arq Bras Cardiol.

[7] Rienstra M, Lubitz SA, Mahida S, Magnani JW, Fontes JD, Sinner MF (2012). Symptoms and Functional Status of Patients with Atrial Fibrillation: State of the Art and Future Research Opportunities. Circulation.

[8] Zimetbaum P, Reynolds MR, Ho KK, Gaziano T, McDonald MJ, McClennen S (2003). Impact of a Practice Guideline for Patients with Atrial Fibrillation on Medical Resource Utilization and Costs. Am J Cardiol.

[9] Ahmed A, Ullah W, Hussain I, Roomi S, Sattar Y, Ahmed F (2019). Atrial Fibrillation: A Leading Cause of Heart Failure-Related Hospitalizations; a Dual Epidemic. Am J Cardiovasc Dis.

[10] Thrall G, Lane D, Carroll D, Lip GY (2006). Quality of Life in Patients with Atrial Fibrillation: A Systematic Review. Am J Med.

[11] Kassianos G, Arden C, Hogan S, Dew R, Fuat A (2013). Current Management of Atrial Fibrillation: An Observational Study in NHS Primary Care. BMJ Open.

[12] Steinberg BA, Piccini JP (2014). Anticoagulation in Atrial Fibrillation. BMJ.

[13] Wu J, Zhang Y, Liao X, Lei Y (2020). Anticoagulation Therapy for Non-valvular Atrial Fibrillation: A Mini-Review. Front Med.

[14] Connolly SJ, Ezekowitz MD, Yusuf S, Eikelboom J, Oldgren J, Parekh A (2009). Dabigatran versus Warfarin in Patients with Atrial Fibrillation. N Engl J Med.

[15] Patel MR, Mahaffey KW, Garg J, Pan G, Singer DE, Hacke W (2011). Rivaroxaban versus Warfarin in Nonvalvular Atrial Fibrillation. N Engl J Med.

[16] Granger CB, Alexander JH, McMurray JJ, Lopes RD, Hylek EM, Hanna M (2011). Apixaban versus Warfarin in Patients with Atrial Fibrillation. N Engl J Med.

[17] Giugliano RP, Ruff CT, Braunwald E, Murphy SA, Wiviott SD, Halperin JL (2013). Edoxaban versus Warfarin in Patients with Atrial Fibrillation. N Engl J Med.

[18] Brasil. Ministério da Saúde (2016). Comissão Nacional de Incorporação de Tecnologias no SUS. Apixabana, Rivoraxabana e Dabigratana em Pacientes com Fibrilação Atrial Não Valvar.

[19] Ruff CT, Giugliano RP, Antman EM, Crugnale SE, Bocanegra T, Mercuri M (2010). Evaluation of the Novel Factor Xa Inhibitor Edoxaban Compared with Warfarin in Patients with Atrial Fibrillation: Design and Rationale for the Effective aNticoaGulation with Factor xA Next GEneration in Atrial Fibrillation-Thrombolysis In Myocardial Infarction Study 48 (ENGAGE AF-TIMI 48). Am Heart J.

[20] Büller HR, Décousus H, Grosso MA, Mercuri M, Middeldorp S, Prins MH (2013). Edoxaban versus Warfarin for the Treatment of Symptomatic Venous Thromboembolism. N Engl J Med.

[21] Bounameaux H, Camm AJ (2014). Edoxaban: An Update on the New Oral Direct Factor Xa Inhibitor. Drugs.

[22] Okumura K, Akao M, Yoshida T, Kawata M, Okazaki O, Akashi S (2020). Low-Dose Edoxaban in Very Elderly Patients with Atrial Fibrillation. N Engl J Med.

[23] Marston XL, Wang R, Yeh YC, Zimmermann L, Ye X, Gao X (2022). Comparison of Clinical Outcomes of Edoxaban versus Apixaban, Dabigatran, Rivaroxaban, and Vitamin K Antagonists in Patients with Atrial Fibrillation in Germany: A Real-World Cohort Study. Int J Cardiol.

[24] Kaatz S, Ahmad D, Spyropoulos AC, Schulman S, Subcommittee on Control of Anticoagulation (2015). Definition of Clinically Relevant Non-Major Bleeding in Studies of Anticoagulants in Atrial Fibrillation and Venous Thromboembolic Disease in Non-Surgical Patients: Communication from the SSC of the ISTH. J Thromb Haemost.

[25] Schulman S, Angerås U, Bergqvist D, Eriksson B, Lassen MR, Fisher W (2010). Definition of Major Bleeding in Clinical Investigations of Antihemostatic Medicinal Products in Surgical Patients. J Thromb Haemost.

[26] Faraoni D, Schaefer ST (2016). Randomized Controlled Trials vs. Observational Studies: Why Not Just Live Together?. BMC Anesthesiol.

[27] Büller HR, Décousus H, Grosso MA, Mercuri M, Middeldorp S, Prins MH (2013). Edoxaban versus Warfarin for the Treatment of Symptomatic Venous Thromboembolism. N Engl J Med.

[28] Chung N, Jeon HK, Lien LM, Lai WT, Tse HF, Chung WS (2011). Safety of Edoxaban, an Oral Factor Xa Inhibitor, in Asian Patients with Non-Valvular Atrial Fibrillation. Thromb Haemost.

[29] Weitz JI, Connolly SJ, Patel I, Salazar D, Rohatagi S, Mendell J (2010). Randomised, Parallel-Group, Multicentre, Multinational Phase 2 Study Comparing Edoxaban, an Oral Factor Xa Inhibitor, with Warfarin for Stroke Prevention in Patients with Atrial Fibrillation. Thromb Haemost.

[30] Yamashita T, Koretsune Y, Yasaka M, Inoue H, Kawai Y, Yamaguchi T (2012). Randomized, Multicenter, Warfarin-Controlled Phase II Study of Edoxaban in Japanese Patients with Non-Valvular Atrial Fibrillation. Circ J.

[31] Li S, Liu B, Xu D, Xu Y (2014). Bleeding Risk and Mortality of Edoxaban: A Pooled Meta-Analysis of Randomized Controlled Trials. PLoS One.

[32] U.S. Food and Drug Administration (2011). E2F Development Safety Update Report [Internet].

[33] Frisullo G, Profice P, Brunetti V, Scala I, Bellavia S, Broccolini A (2020). Prospective Observational Study of Safety of Early Treatment with Edoxaban in Patients with Ischemic Stroke and Atrial Fibrillation (SATES Study). Brain Sci.

[34] de Groot JR, Weiss TW, Kelly P, Monteiro P, Deharo JC, de Asmundis C (2021). Edoxaban for Stroke Prevention in Atrial Fibrillation in Routine Clinical Care: 1-Year Follow-Up of the Prospective Observational ETNA-AF-Europe Study. Eur Heart J Cardiovasc Pharmacother.

[35] Kirchhof P, Pecen L, Bakhai A, de Asmundis C, de Groot JR, Deharo JC (2022). Edoxaban for Stroke Prevention in Atrial Fibrillation and Age-Adjusted Predictors of Clinical Outcomes in Routine Clinical Care. Eur Heart J Cardiovasc Pharmacother.

[36] Suarez-Kurtz G (2010). Pharmacogenetics in the Brazilian Population. Front Pharmacol.

