# Supplementary material for: Desenho de Estudo de um Estudo Observacional Brasileiro sobre o uso de Edoxabana em Pacientes com Fibrilação Atrial (EdoBRA)
Source: Arq Bras Cardiol. 2024 Apr 12;121(3):e20230392. [Article in Portuguese] doi: 10.36660/abc.20230392 (PMC12092049; doi:10.36660/abc.20230392)
Supplement: Translation

# Translation: Study Design of a Brazilian Observational Study of Edoxaban in Patients with Atrial Fibrillation (EdoBRA)

**DOI:** 10.36660/abc.20230392i

**Authors:** Dalton Bertolim Précoma, Rafael Paletta da Silva, Allyson Nakamoto, Viviane Mariz Omar, Danilo Lopes, José Francisco Kerr Saraiva

**Affiliations:** 1 Curitiba PR Brazil Hospital Angelina Caron, Curitiba, PR – Brazil; 2 São Paulo SP Brazil Daiichi Sankyo Brasil Farmacêutica, São Paulo, SP – Brazil; 3 Campinas SP Brazil Pontifícia Universidade Católica de Campinas, Campinas, SP – Brazil

**Keywords:** Oral Anticoagulants, Edoxaban, Observational Study, Safety, Efficacy

## Abstract

**Background:**

Clinical trials showed the safety of Edoxaban, a non-vitamin K-dependent oral anticoagulant (NOAC), and its efficacy to prevent stroke and systemic embolism in non-valvular atrial fibrillation (NVAF) patients and also to prevent and treat venous thromboembolism. However, additional research is needed to evaluate the safety and effectiveness of Edoxaban in a real-world scenario in the Brazilian population.

**Objective:**

In order to understand the risks and benefits of Edoxaban use in routine clinical settings, the EdoBRA study is being conducted to gain insight into the safety and effectiveness of Edoxaban use in non-preselected patients with NVAF in Brazil.

**Methods:**

The EdoBRA study is a multicenter, prospective, observational study conducted in 36 sites in Brazil. NVAF patients ≥ 18 years treated with commercially available Edoxaban who initiated treatment for at least 14 days and no longer than 90 days prior to enrollment, and who are not simultaneously participating in any interventional study are eligible for this study. Seven hundred patients are planned to be enrolled and one-year of follow up, with data collections expected at baseline and 3, 6, and 12 months after the study enrollment. The primary safety objective is ISTH Clinically Relevant Bleeding, and the secondary effectiveness objective focuses on relevant cardiovascular outcomes related to NVAF.

**Conclusion:**

EdoBRA observational study will generate relevant additional information about NOAC Edoxaban on various aspects of patient management in routine care, such as its safety and effectiveness profile in patients with NVAF in Brazil.

## Introduction

Atrial fibrillation (AF) is the most common arrhythmia reported in clinical practice, with approximately 2,5% of adults aged 20 years or older being affected.^1 , 2^Like other parts of the world, there is a growing prevalence of AF in older adults in Latin America, characterized by a silent form at first detection to a long-standing persistent up to permanent form.^2 – 4^Most cases are non-valvular AF (NVAF), which is usually associated with increased morbidity and mortality. The main problems associated with it are the risk of stroke, bleeding, and death from cardiovascular causes.^5 , 6^Hospitalizations due to AF or its complications (aggravation of heart failure, thromboembolic complications, and acute arrhythmia management) account for one-third to two-thirds of all admissions for cardiac arrhythmias.^7 – 9^Patients with NVAF have a significantly poorer quality of life compared with healthy controls, the general population, or patients with coronary heart disease.^10^The annual risk of stroke is five to six times greater in patients with AF than in people with a normal heart rhythm.^11^

The goal of NVAF treatment with anticoagulants is to prevent stroke and/or systemic embolism.^12 , 13^However, one important complication of anticoagulation therapy, which could also affect patient adherence and persistence, is bleeding. Thus, different anticoagulants, such as the non-vitamin K-dependent oral anticoagulant (NOAC), emerged to treat patients with NVAF and mitigate this gap. NOAC enables the administration of fixed doses without the need for routine monitoring of coagulation, dose adjustment requirements, and the substantial risk of bleeding, as commonly described by older anticoagulants such as warfarin. Currently, four NOACs have been approved by several regulatory authorities globally, including the Brazilian Health Regulatory Agency (ANVISA), and are available for clinical use: direct thrombin inhibitor, dabigatran; and the oral factor Xa inhibitors, rivaroxaban, apixaban, and Edoxaban. These new oral anticoagulants are as safe and effective as warfarin for the prevention of stroke and systemic embolism in patients with AF,^14 – 17^and their routine clinical use has been described in the literature.^18^

Most randomized clinical trials adopted restricted eligibility criteria, having either explicitly excluded patients or enrolled only relatively healthy individuals with fewer comorbidities or functional impairments. Thus, prospective registries of real-world evidence can add relevant information about the effectiveness of these drugs in the Brazilian population. The EdoBRA study is designed to prospectively collect detailed information on the use of Edoxaban in real-world clinical settings in non-preselected NVAF patients in Brazil. Real-world data would provide us with a greater understanding of NVAF treatment pathways and broaden our insight into the patient population.

### Edoxaban

Edoxaban is an orally administered anticoagulant that inhibits coagulation factor Xa. It was approved for marketing in Brazil by ANVISA in March 2018 for the following indications: risk reduction of stroke and systemic embolic events (SEE) in adult patients with non-valvular atrial fibrillation (NVAF) and for the treatment of venous thromboembolism (VTE), including deep vein thrombosis (DVT) and pulmonary embolism (PE), and the prevention of recurrent VTE (DVT and/or PE).

Its approval was based on the results of the ENGAGE AF-TIMI 48 trial, a multinational, randomized, double-blind, double-dummy, non-inferiority study comparing the efficacy and safety of two dosing regimens of once-daily Edoxaban to dose-adjusted warfarin (target international normalized ratio [INR] 2.0 to 3.0) in adult patients (N = 21,105) with NVAF at moderate-to-high risk of stroke. Edoxaban, both high-dose (60/30 mg) and low-dose (30/15 mg) once daily exhibited similar efficacy for the prevention of stroke or SEE and significantly lower rates of bleeding and death from cardiovascular causes compared with well-managed warfarin in patients with AF.^17 , 19^

The Hokusai-VTE study (N = 8,292) was also pivotal for the approval of Edoxaban. The study found that patients with symptomatic VTE had a flexible treatment duration of 3–12 months and found that following initial heparin, Edoxaban 60 mg once daily was non-inferior to dose-adjusted warfarin (INR 2.0–3.0) for the prevention of recurrent VTE, and also had a significantly lower risk for the composite of major or non-major clinically relevant bleedings (primary safety outcome).^20 , 21^

Another pivotal study is the ELDERCARE-AF trial (N = 984). The objective was to assess the prevention of stroke in very elderly patients with AF. After this multicenter, randomized, double-blind, placebo-controlled, event-driven trial, researchers found that a once-daily 15 mg dose of Edoxaban was superior to placebo in preventing stroke or systemic embolism (2.3% in Edoxaban and 6.7% in placebo) and did not result in a significantly higher incidence of major bleedings when compared to placebo.^22^

A large European real-world study reporting two-year outcomes on Edoxaban therapy found no additional safety signals and/or event rates than those observed in ETNA-AF after one year and in ENGAGE AF-TIMI 48 trial. These outcomes reinforce the outcomes found in Edoxaban clinical trials and the license recommendations in a real-world scenario. Additionally, real-world comparative studies indicated better effectiveness findings in patients receiving Edoxaban treatment than other therapies, such as phenprocoumon or even other NOACs.^23^

Since its availability in Brazil, thousands of patients have been treated with this NOAC. Still, it is also important to assess its performance in a routine clinical scenario in the Brazilian population. In this paper, we described the design and methodology of EdoBRA study, including the common parameters (i.e., the so-called "core data"), the observational periods, and other technical details such as the consistency of terminology.

To help understand the risks and benefits of Edoxaban use in a clinical setting close to regular clinical practice, we conducted this non-interventional study (NIS) to gain insight into the safety and effectiveness aspects in non-preselected patients with NVAF treated with Edoxaban in Brazil.

The key goal of this study is to evaluate the one-year safety of Edoxaban regarding the occurrence of Clinically Relevant Bleeding (Clinically Relevant Non-Major Bleeding and Major Bleeding) in patients with NVAF. It is also expected that the data collected during the study provide an overview of effectiveness outcomes in preventing strokes (ischemic and hemorrhagic), SEE, transient ischemic attack (TIA), major adverse cardiovascular events (MACE, composite endpoint of non-fatal MI, non-fatal stroke, non-fatal SEE, and death due to CV cause or bleeding), VTE, acute coronary syndrome (ACS), and hospitalizations related to cardiovascular (CV) condition.

Thus, real-world evidence data in routine clinical practice using Edoxaban for up to one year will be collected and evaluated in 700 patients with NVAF, treated by specialized as well as non-specialized physicians in different hospital-based centers in Brazil.

## Methods

### Study design

The EdoBRA study, sponsored by Daiichi-Sankyo Brazil, is a multicenter, prospective, observational (non-interventional - NI) study conducted in Brazil. It was designed as a NIS because it includes patients who received Edoxaban prescriptions with no prescription influence for inclusion in the study. Principal investigators may not include patients to whom they have prescribed Edoxaban. Only patients referred by other physicians are eligible.

Enrollment and data collection commenced in October 2019 and completed in February 2023. The routine clinical practice data documentation is expected to include four different time points: at baseline, after 3 (±2), 6 (±2), and 12 (±1) months of study enrollment (one year of follow-up). Even patients discontinuing treatment with Edoxaban will be followed up to one year. Patients’ visits are conducted according to regular clinical care and are not influenced by the foreseen documentation data time point. As an observational study, it does not require a specific visit schedule; thus, the frequency of data collection is mostly consistent with the usual regular clinical care visits of patients with NVAF to health services, according to clinical practice. The study design is shown in Figure 1 .

**Figure 1:** Patient follow-up flowchart. FUP: Follow-up. Disclaimer: As it is a non-interventional, real-world study, the visits are not scheduled. Only routine clinical practice data will be documented.

### Eligibility and exclusion criteria

The EdoBRA study planned to include approximately 700 patients with an established diagnosis of NVAF (all types of NVAF were considered) and treated with Edoxaban according to the local label. Eligible patients are those ≥ 18 years diagnosed with NVAF who initiated Edoxaban treatment for at least 14 days and no longer than 90 days prior to enrollment. Patients must consent to participate and must not participate in any interventional study simultaneously; however, simultaneous participation in any other NIS/registry is allowed. No other explicit exclusion criteria were adopted to minimize selection bias and to allow for documentation of routine clinical practice as much as possible in this NIS.

### Assessments

The primary objective of this study is to evaluate the safety of Edoxaban in regular clinical care settings. The safety profile will be measured by the frequency of the Major Bleeding or Clinically Relevant Non-Major (CRNM) bleeding according to the International Society of Thrombosis and Haemostasis (ISTH) criteria in patients with NVAF during one year of follow-up.

A clinically overt bleeding event (i.e., bleeding that is visualized by examination or radiologic imaging) that meets at least one of the following criteria: a) fatal bleeding; b) symptomatic bleeding in a critical area or organ such as retroperitoneal, intracranial, intraocular, intraspinal, intra-articular, pericardial, or intramuscular with compartment syndrome.

Based on the ISTH classification, a non-major bleeding event but clinically relevant (CRNM) was defined according to the following assumptions: over-bleeding requires medical intervention by a healthcare professional; one that leads to hospitalization or increased level of care; one that prompts a face-to-face evaluation (i.e., not remote care) but that does not fulfill the criteria for a major bleeding event.

In case patients present other overt bleeding events that do not meet the criteria of a major bleeding event or a clinically relevant non-major bleeding event (e.g. epistaxis that does not require medical attention), it should be classified as a minor bleeding event. In addition, all bleeding events captured during the data collection should be assessed according to the ISTH bleeding definitions.^24 , 25^If the patient presents other events, such as reduced hemoglobin levels without bleeding, it should be considered a non-bleeding event. The difference between the ISTH bleeding events classification is described in Figure 2 .

**Figure 2:** Events that define ISTH Major Bleeding Events and ISTH Clinically Relevant Non-Major Bleeding Events. ISTH: International Society of Thrombosis and Hemostasis.

The secondary objective includes the effectiveness of Edoxaban in avoiding events like strokes (both ischemic and hemorrhagic), SEE, TIA Major Adverse Cardiovascular Events (MACE), VTE, ACS, composite endpoint of non-fatal events –MACE (non-fatal MI, non-fatal stroke, non-fatal SEE, and death due to CV cause or bleeding), and hospitalizations causes (overall, related to CV condition or related to bleeding condition). Finally, the safety profile regarding other adverse events that do not fill the primary outcome criteria will also be assessed.

Thus, all safety measures that occur between enrollment and final follow-up will be recorded, including all adverse events. Additionally, baseline demographic and clinical characteristics will be recorded for all patients. AF history and diagnosis, details of Edoxaban prescribing information, diagnostic/therapeutic procedures, and clinical outcomes will be documented at baseline and updated with any new information for each procedure recorded up to a one-year period (±1 month).

### Data management

All data elements will be collected from information routinely recorded in the patient files/medical records. There are no recommendations to alter routine clinical practice, nor should it be changed due to this NIS. No specific visits or examinations, laboratory tests, or procedures are mandated as part of this study. Since neither the Principal Investigator nor Sub-Investigators will be able to include patients to whom they have prescribed Edoxaban, the study team will be primarily accountable for data collection of referred patients. Access to source documents (e.g., medical records) is mandatory to capture data in the electronic Clinical Data Capture (eCRF). Source data must also be available for site monitoring activities.

An Eletronic Data Capture (EDC) system is used for data capture. All clinical data is documented using an eCRF tool designed specifically for this study. Predefined variables and definitions are used, and free text field documentation was limited as much as possible. In the start-up study phase, a data management plan has been created describing the entire data collection process. Automatic plausibility checks and manual data review at data entry are performed to avoid potential mistakes and to allow corrections or confirmation by the sites. In addition, the eCRF tool also presents an automatic reporting system for adverse events. To comply with the adverse event reporting deadlines, the eCRF tool sends an automatic message to monitors and the pharmacovigilance team every time the data abstractor enters information related to a serious adverse event or an adverse drug reaction.

Medical history, clinical events and descriptions, and adverse events/drug reactions will be coded using the Medical Dictionary for Regulatory Activities (MedDRA). Clinical events are based on physician diagnosis and assessment. Medications/drugs will be coded using the World Health Organization Drug Dictionary Enhanced (WHO-DDE).

The number of missing cases will be reported for each variable of interest in the analysis. The flow of study participants through the entire study period will be characterized in terms of the number of subjects who completed the study, withdrew from it (with reasons described), or died. Additionally, if a patient's treatment on Edoxaban is permanently discontinued for any reason at any time after the baseline data collection point, the investigator needs to document this event in the eCRF accordingly.

All Serious adverse events (SAEs) and all adverse drug reactions (ADRs) must be entered in the respective data collection section of the eCRF (electronic report form) as soon as the physician becomes aware of it. Non-SAEs that are not related to Edoxaban will not be documented. Adverse events will be reported according to the national requirement and local law in Brazil.

### Sample size and statistical analysis

No formal sample size consideration has been performed for this study. The study aims to enroll 700 patients in 36 sites. It is reasonable to estimate that 2-9% of patients will present at least one clinically relevant bleeding event during follow-up, which will allow descriptive analysis and incidence of bleeding estimations to be conducted.

As this study is observational and descriptive, no statistical hypothesis testing is intended. Therefore, only descriptive statistics will be performed to describe all collected data and generally limited to frequency tables or summary statistics (e.g., means ± standard deviation or medians ± quartiles), e.g., for demographic data. The incidence of clinically relevant non-major bleeding and major bleeding events will be calculated based on the proportion of patients presenting bleeding events in the follow-up period. Data analyses for follow-up data are planned at three and six months, followed by the baseline and final analyses of complete follow-up data up to one year (±1 month). The number of subjects with at least one event will be presented (n [%]) separately for each type of clinical event. ADRs will be summarized based on MedDRA.

In addition, where applicable, the index date for the study analyses will be the Edoxaban start date. All statistical analysis will be performed using Python version 3.6.9.

### Quality control

This study has been conducted according to the rules of ‘Good Pharmacoepidemiology Practice’ (GPP) and relevant local regulations. Related quality control mechanisms have been performed accordingly.

On-site monitoring will be performed in a minimum of 30% randomly selected patients. During on-site monitoring, the monitor will verify 100% of informed consent documentation and perform source data verification against the selected patient's medical records. Particular attention will be paid to the completeness and correctness of safety data during monitoring activities. Validation and quality control will be performed to ensure that the reported data are as accurate and complete as possible in a routine clinical care setting and that the conduct of the EdoBRA study is compliant with the plans and local applicable regulatory requirements.

## Discussion

Observation research has a pivotal role in confirming the effectiveness and safety of recently approved interventions across a range of patients with different characteristics (demographics, comorbidities, health care access, and others) treated in a clinical environment in which control of patient therapy is often not as strict as in a clinical trial. They may also identify adverse effects associated with the use of pharmaceuticals that were not anticipated based on research conducted in support of the drug approval process. The EdoBRA study is a multicenter, prospective, observational (non-interventional) study conducted in Brazil to evaluate the safety and effectiveness of Edoxaban in patients with NVAF. The design of EdoBRA core data addresses key questions about the clinical usage of Edoxaban, providing evidence of the clinical characteristics and important outcomes in routine clinical usage, allowing information to be complemented with the already published RCT.^26^

Previously, some RCTs have been performed to assess bleeding risk. The ENGAGE AF-TIMI 48 trial,^17^for example, was a large trial that enrolled 21,105 subjects with moderate-to-high AF risk comparing Edoxaban and Warfarin. Subjects receiving Edoxaban were associated with significantly lower rates of bleeding and death by cardiovascular causes. In agreement with the ENGAGE study, other studies found lower rates of bleeding in patients with VTE^27^or NVAF.^28^Additionally, other RCTs given 30 and 60 mg of Edoxaban, as well as a pooled meta-analysis of five prospective RCTs totalizing 311,262 patients, indicated that Edoxaban seems to have a favorable safety profile in comparison with warfarin regarding bleeding and mortality risk.^29 – 31^Although Edoxaban achieved a good performance in these studies, there are concerns about the potential for bleeding in the clinical practice that need to be investigated with real-world studies.^31^Although Randomized Clinical Trials (RCT) are the gold standard model for demonstrating the safety and efficacy of a new drug. The inclusion and exclusion criteria in this type of study select certain patients and do not represent the whole population. Given the natural limitations of RCTs, regulatory agencies around the world understand that NISs are supportive and complement RCT data.^32^

Some observational studies about Edoxaban have been developed in the past years. The STATES Study^33^evaluated the occurrence of symptomatic bleeding from acute cardioembolic stroke in patients who received early treatment with Edoxaban in a small elderly population (n = 75) for three months. Outcomes indicated that Edoxaban is safe, but further studies with a bigger population and a longer follow-up period are recommended. A large observational and prospective study – ETNA-AF, conducted in ten European countries evaluated the safety and effectiveness of 30 mg and 60 mg of Edoxaban in routine care of 13,092 patients for one year. They concluded that the rates of stroke, systemic embolism (0.82%/year), and major bleeding (1.05%/year) were low in the unselected cohort,^34^reinforcing the safety of Edoxaban. Furthermore, a two-year follow-up period of the ETNA-AF was recently published, providing real-world evidence for the long-term safety and effectiveness of Edoxaban. The outcomes reinforce the low rates of stroke (0.70%) and major bleeding (0.97%) in patients with AF and pointed out the high adherence to the treatment with almost 70% of patients under Edoxaban treatment at the two-year follow-up.^35^The age-adjusted risk predictors analysis showed that, at baseline, history data such as TIA and the recalculated eCrCl were the strongest predictors of ischemic stroke and major bleeding, respectively. Besides the strong evidence, data on its effectiveness and safety in routine clinical care is still limited in the Brazilian population.^35^Therefore, post-authorization studies, such as EdoBRA, can provide important additional information on real-world aspects of patient management in routine care in Brazil.

This specific study develops an important and unique role in evaluating the real-world effectiveness and safety in regular clinical care, assessing the occurrence of ISTH Clinically Relevant Bleeding within one year of follow-up in ~700 patients in Brazil. Questions regarding the existence of regional usage patterns and/or patient characteristics, how they affect outcomes, and how the timing of clinical events relative to the start of dosing, dosing interruption, or any situation related to the treatment scheme can be examined. In addition, it should be considered that the Brazilian population is characterized by a great ethnic diversity and racial fusions not easily found in other regions of the world,^36^which also justifies better knowledge of the behavior of anticoagulant drugs through real-life records.

As this study aims to collect and evaluate real-world evidence, some limitations common to non-interventional studies apply, and some aspects need to be considered. Effective observational research must recognize the potential for bias and attempt to minimize it both in the design and analysis stages, as well as accurately describe its limitations. The lack of randomization may introduce selection bias, especially for comparing outcomes. As the study is non-interventional, only data from the clinical routine treatment can be obtained. Therefore, some information may be missing or unavailable. The study design chosen for this study does not allow causality conclusions but rather for interferences and indication of pathways for further investigation. As a result of the descriptive nature of the study, results must be interpreted carefully to avoid direct comparisons among different groups. Although selected sites were in different regions of Brazil, most of them were in the Southeast, Northeast, and South regions. Thus, a decrease in terms of representativeness of the Brazilian population may occur.

## Conclusion

EdoBRA will provide a comprehensive data set to support the safety and clinical utility of Edoxaban in patients with AF in routine practice. Also, the study will provide important insights into the management of adverse events occurring in patients receiving Edoxaban therapy, as well as indicators that can contribute to maximizing patient satisfaction and optimizing real-world outcomes.
